# Keap-NRF2 signaling contributes to the Notch1 protected heart against ischemic reperfusion injury via regulating mitochondrial ROS generation and bioenergetics

**DOI:** 10.7150/ijbs.63297

**Published:** 2022-02-07

**Authors:** Hua Xu, Xiao-dan Wan, Rong-rong Zhu, Jin-long Liu, Ji-chun Liu, Xue-liang Zhou

**Affiliations:** 1 Department of Cardiac Surgery, The First Affiliated Hospital, Nanchang University, Nanchang, Jiangxi 330006, China.; 2 Department of Electrocardiogram, The First Affiliated Hospital, Nanchang University, Nanchang, Jiangxi 330006, China.; 3 Department of Obstetrics and Gynecology, Gaoxin Hospital of the First Affiliated Hospital of Nanchang University, Nanchang, China.; 4 Zhangjiang Institute, Fudan University, Shanghai, China.

**Keywords:** NRF2, Notch1, mitochondrial ROS, mitochondrial bioenergetics, ischemic reperfusion injury

## Abstract

Myocardial ischemia/reperfusion (I/R) injury is recognized as the leading cause of death worldwide. However, the molecular mechanisms involved in this process are still not fully understood. We previously reported that the combined action of Notch1 and Keap1-NRF2 signaling pathway can significantly increase the activity of cardiomyocytes, inhibit the apoptosis of cardiomyocytes, reduce the formation of reactive oxygen species, and improve the antioxidant activity in neonate rat myocardial cells. However, the regulatory mechanism of Notch1 signaling pathway on the NRF2 signaling pathway and its actual role on I/R injury are still unclear. Herein, we found that Keap-NRF2 signaling is activated by Notch1 in RBP-Jκ dependent manner, thus protects the heart against I/R injury via inhibiting the mitochondrial ROS generation and improves the mitochondrial bioenergetics in vitro and in vivo. These results suggest that Keap-NRF2 signaling might become a promising therapeutic strategy for treating myocardial I/R injury.

## Introduction

Myocardial ischemia/reperfusion (I/R) injury plays an important role in coronary heart disease (CHD), which is recognized as the leading cause of death worldwide^1^-^4^. However, the molecular mechanisms involved in this process are still not fully understood. Therefore, there is an urgent need to explore the potential molecular mechanism of myocardial I/R injury.

Notch signaling pathway has a protective effect on myocardial ischemia-reperfusion injury^5^. Notch1 is a transmembrane protein belonging to the Notch family. By binding to Notch ligand, Notch1 is cleaved by proteases such as γ-secretase to release the intracellular domain N1ICD, which translocate to the nucleus and acts as a transcription cofactor to regulate the expression of target genes^6^-^9^. Recently, we reported that Notch1 regulates the dynamic balance between mitochondrial fusion/division and mitochondrial autophagy in I/R injury cardiomyocytes, and provides protection for cardiomyocytes^10^,^11^. In addition, the combined action of Notch1 and Keap1-NRF2 signaling pathway can significantly increase the activity of cardiomyocytes, inhibit the apoptosis of cardiomyocytes, reduce the formation of reactive oxygen species, and improve the antioxidant activity in neonate rat myocardial cells^12^,^13^. However, the regulatory mechanism of Notch1 signaling pathway on the Nrf2 signaling pathway and its actual role on I/R injury are still unclear.

Herein, we demonstrate that Keap-NRF2 signaling is activated by Notch1 in RBP-Jκ dependent manner. Moreover, it protects the heart against I/R injury via inhibiting the mitochondrial ROS generation and improves the mitochondrial bioenergetics in vitro and in vivo. These results suggest that Keap-NRF2 signaling might become a promising therapeutic strategy for treating myocardial I/R injury.

## Materials and Methods

### Animal care

Animals used in this study were maintained in accordance with the Guide for the Care and Use of Laboratory Animals (Publication 85-23, National Institutes of Health, Bethesda, MD), and all procedures were approved by the First Affiliated Hospital of Nanchang University (Nanchang, Jiangxi, China).

### Isolation and culture of ventricular myocytes

Left ventricular myocytes were isolated from adult rat hearts using a standard enzymatic method as previously described^11^. Briefly, the freshly separated heart was successively infused with a nominally Ca^2+^ free Tyrode's solution containing collagenase II (240 U/mL, Worthington Biochemical, USA) and protease (0.1 mg/mL, Sigma, USA) for 20 minutes. The cell suspension extracted from the left ventricle was rinsed with Tyrode's solution, and the Ca^2+^ concentration was gradually increased up to 1.25 mM. The isolated cells were added to M199 culture medium (Sigma, USA) with L-carnitine (2 mmol/L), N-2-mercaptopropyl glycine (5 mmol/L), taurine (5 mmol/L), insulin (0.1 mmol/L), 2.5% fetal bovine serum (Gibico, USA) and penicillin streptomycin (100 IU/mL).

### Simulated H/R in isolated cardiomyocytes

A cellular model of simulated H/R (20-min/30-min) in ventricular myocytes was used as previously described^13^. Briefly, the primary left ventricular myocytes were cultured in anoxic solution in an anoxic incubator (95% N_2_/5% CO_2_) for 3 hours. The hypoxia solution was then replaced by reoxygenation solution and cultured in a high-oxygen incubator (95%O_2_/5%-CO_2_) for 3 hours.

### Construction and infection of recombinant adenoviruses

Recombinant adenoviruses expressing Rat N1ICD/NRF2 complementary DNA (cDNA) and shN1ICD/shNRF2 shRNA were prepared using the pAdEasyTM vector system (Qbiogene) as described previously. Primary cardiac myocytes were infected with adenoviral particles at the multiplicity of infection of 100.

### Cell viability assay

The cell viability of adult cardiomyocytes was detected with CCK-8 assay (Dojindo) as described previously^13^. Briefly, primary left ventricular myocytes were inoculated in 96-well plates at a rate of 10^3^ cells/well, cultured in 100 μ L M199 medium for 24 h, and treated as indicated. Finally, cell viability was measured using the CCK-8 kit according to the manufacturer's instructions. A microplate reader (Thermo, USA) was used to measure the absorbance at 450nm. All the experiments were repeated three times independently.

### NRF2 promoter activity assay

The normal, Notch1 or RBP-Jκ over-expressed cardiomyocytes were incubated in 24-well plates with 2×10^4^ cells per well. These cells were then transfected with pGL3-NRF2-WT or pGL3-NRF2-Mut (RBP-Jκ binding site mutant). After 48 h, the cells were lysed with a passive lysis buffer (Promega, Madison, WI, USA) and the luciferase reporting assay (Promega) was calculated.

### TUNEL staining

Apoptosis rates in cultured cardiomyocytes and mouse heart tissue sections were analyzed by TUNEL staining using the in-situ cell death detection kit (Roche Applied Science) according to the manufacturer's protocol. In summary, the slides were incubated with TUNEL reaction mixture, apoptotic cells were labeled, and the total number of cells was determined by DAPI staining. The slides were viewed under a Zeiss LSM800 laser scanning confocal microscope (Zeiss, Wetzlar, Germany). Apoptosis rate was calculated as the percentage of TUNEL positive cells in the total number of DAPI stained cells. All the experiments were repeated three times independently.

### Measurement of cardiac troponin I (cTnI), lactate dehydrogenase (LDH) and CK levels creatine kinase-MB (CK)

The coronary effluent and culture medium of cardiomyocytes after reperfusion were placed in a thermostatic chamber. Electrochemiluminescence immunoassay was used to detect the level of cTnI, LDH and CK according to the instructions of the kit (Roche, Germany). All the experiments were repeated three times independently.

### ROS detection

The cardiomyocytes were seeded into 6-well plates at 1×10^6^ cells/well. After H/R, cells were incubated with DCFH-DA (10 μmol/L) at 37 °C for 20 min and ROS were detected by a FACs (BD Biosciences, USA). All the experiments were repeated three times independently.

### Measurement of mitochondrial ROS

To measure mitochondrial ROS, cells were incubated with Mito-SOX dye (TMRE, Molecular Probes) for 20 min, and then detected under a Zeiss LSM800 laser scanning confocal microscope (Zeiss, Wetzlar, Germany). All the experiments were repeated three times independently.

### Measurement of glutathione (GSH) level, superoxide dismutase (SOD) and NADPH oxidases activity

The measurement of GSH content, SOD, and NADPH oxidases activity are performed according to the test kit instructions (Nanjing Kaiji Bio, Nanjing, China). All the experiments were repeated three times independently.

### Seahorse analysis

As previously described, mitochondrial metabolic flux was tested in adult cardiomyocytes. Briefly, cardiomyocytes were inoculated into XF24 hippocampal plate coated with laminin at a density of 10^4^ cells per well and cultured overnight in BCAA-free substrate-restricted medium, then analyzed. OCR and ECAR were determined using the Seahorse XF24 extracellular flux analyzer (Seahorse Bioscience, North Billerica, MA, USA). All the experiments were repeated three times independently.

### Western blot analysis

The adult cardiomyocytes were lysed in cell lysis buffer (Beyotime Institute of Biotechnology) at 4°C. Protein samples were separated by 8%‐10% SDS‐PAGE, then transferred to nitrocellulose membranes (Millipore). Membranes were incubated with primary antibodies overnight at 4°C, followed by incubation with secondary antibodies at room temperature for 1 hour. The fluorescent signals were detected using enhanced chemiluminescence by ImageQuant LAS4000 (GE). All the experiments were repeated three times independently.

### Real-time PCR

Total RNA was extracted from frozen heart tissues or cultured cells and RNA reverse-transcription were performed as we previously described. RT-PCR was conducted using a SYBR Green Master Mix (Cowin, Beijing, China). All the experiments were repeated three times independently.

### RBP-Jκ and NRF2 promoter luciferase reporter assay

Commercial RBP-Jκ luciferase reporter kit from SA Biosciences Qiagen was used to determine the effect of Notch1 or RBP-Jκ over-expression on transcriptional activity of RBP-Jκ. Cardiomyocytes cells were transfected with the reporter construct using Lipofectamine 3000 (Thermo Fisher). Twenty-four hours after transfection, cells were harvested using lysis buffer. Samples were centrifuged, and 20 μL aliquot was used for measurement of dual luciferase activity using a luminometer.

### Immunofluorescence analysis

Immunofluorescence assay was carried out as described above. The muscle cells fixed with paraformaldehyde were treated with 0.5% Triton X-100 PBS for 15 minutes. The myocytes were then immunostained with anti-KEAP1 (1:100) and anti-Cullin3 (1:100, Santa Cruz, USA) antibodies. After washing with PBS, FITC-conjugated anti-rabbit IgG, PE-conjugated anti-mouse antibody or anti-goat IgG secondary antibody (1:2000, Jackson, USA) were stained for 2 h, respectively. The stained cells were observed under a Zeiss LSM800 confocal microscope (Zeiss, Heidelberg, Germany). All the experiments were repeated three times independently.

### I/R injury model in Langendorff-perfused rat hearts

I/R injury model in Langendorff-perfused rat hearts was performed as previously^12^. In brief, the rats were anesthetized with sodium pentobarbital (45 mg/kg I.P.), the hearts were rapidly resected-and the Krebs-Henselite (K-H) solution was infused at 37 ° C using a Langendorff apparatus at a constant pressure of 80 mm Hg as described in the previous paper. A water-filled latex balloon was connected to a pressure sensor (Gould P23DB, AD instrument) and inserted into the left ventricular cavity to achieve a stable LVEDP of 5-10mmHg during the initial equilibrium. After balanced perfusion, the heart was ischemia-free for 30 minutes, followed by another 45 minutes of perfusion. LVDP and ±dp/dt Max were evaluated using PowerLab system (AD instrument).

### *In vivo* adenoviral gene delivery

The surgical procedures and adenoviral delivery were carried out as described^12^. Briefly, the rats were anesthetized and a 26-gauge needle containing 100 μL of diluted adenoviruses (1× 10^10^ pfu/mL) or saline was advanced from the apex of the left ventricle to the aortic root. The aorta and main pulmonary arteries were clamped for 10 s distal to the site of the injector and solution injected, and then the chest was closed.

### Statistical analysis

Data are expressed as mean ±SEM. Statistical significance was determined by multiple comparisons or repeated measures using analysis of variance or repeated analysis of variance. The student t test was used to estimate the significant difference between the two averages. P < 0.05 was considered statistically significant.

## Results

### NRF2 overexpression impairs the H/R induced mitochondrial ROS generation in adult cardiomyocytes

**Figure 1:**
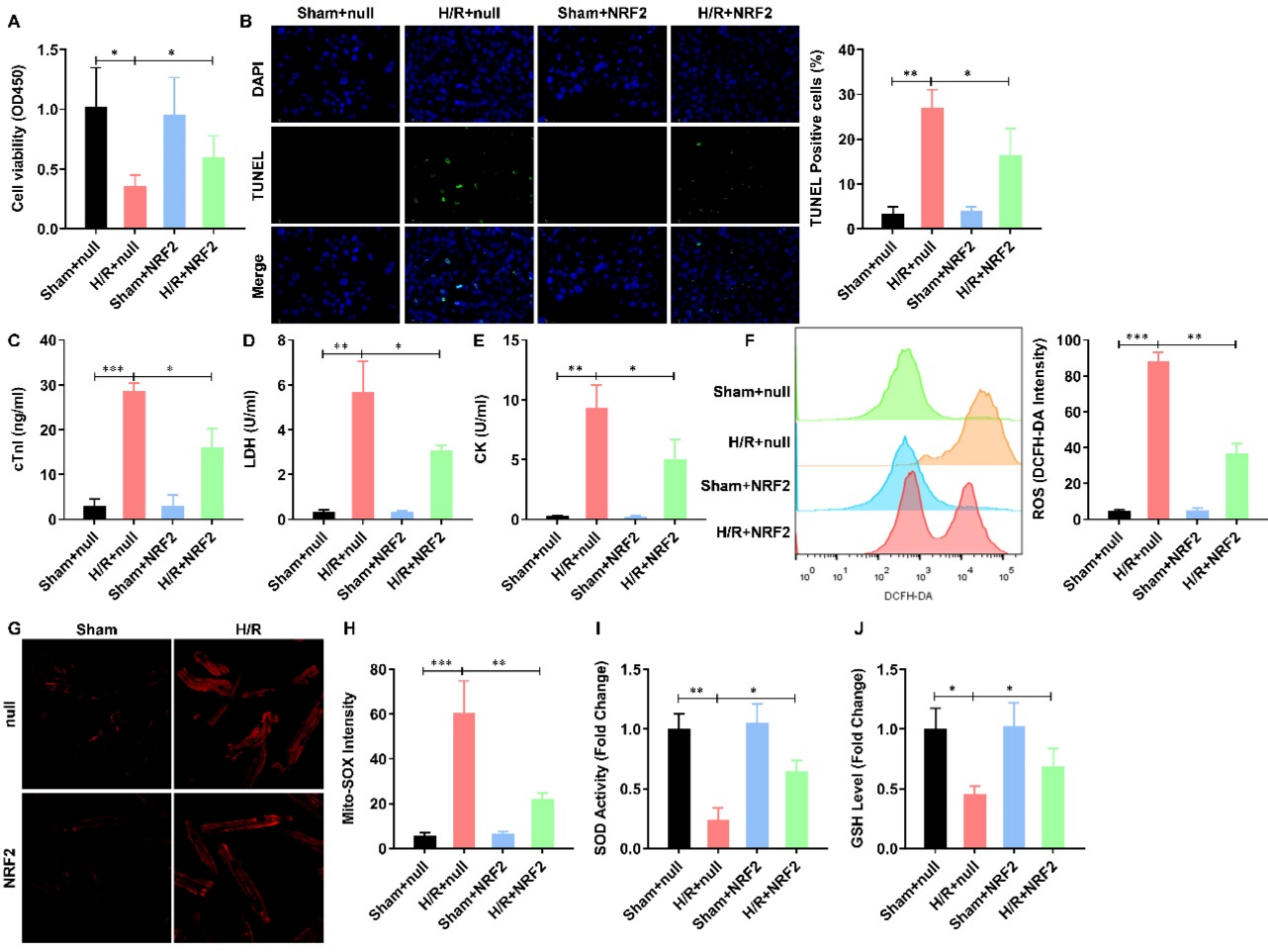
** NRF2 overexpression impairs the H/R induced mitochondrial ROS generation in adult cardiomyocytes.** (A) The cellular viability was detected by CCK-8 assay; (B) The percentage of apoptotic cardiomyocytes was analyzed by TUNE staining; (C) The level of cTnI in the culture medium were also detected at the end of the reperfusion to assess myocardial injury; (D) The activity of LDH and (E) CK were evaluated by Electrochemiluminescence immunoassay; (F) The total cellular and (G) mitochondrial ROS were analyzed by DCFH-DA and Mito-SOX assay; (H) The levels of SOD and (I) GSH were analyzed by ELISA Kit. N=3; *P<0.05, **P<0.01 verses indicated group.

To illustrate the effect of NRF2 on the H/R injured cardiomyocytes, we overexpressed NRF2 in the isolated adult cardiomyocytes by adenovirus. The cell viability was significantly decreased by H/R treatment. NRF2 over-expression did not affect the cell viability, but dramatically attenuated the effect of H/R on adult cardiomyocytes (**Figure 1A**). As apoptosis is the major pathophysiological process underlying liver ischemia/reperfusion (I/R) injury. We then analyzed the H/R induced apoptosis by TUNEL staining, the percentage of H/R induced apoptotic cardiomyocytes was similarly decreased by NRF2 over-expression (**Figure 1B**). Considering cTnI is 100% tissue-specific for myocardial damage and is an excellent marker for myocardial injury, the cTnI levels in the culture medium were also detected at the end of the reperfusion to assess myocardial injury. As shown in **Figure 1C**, the H/R elevated cTnI level was dramatically attenuated by NRF2 over-expression. Consistently, the activity of LDH (**Figure 1D**) and CK (**Figure 1E**) that were also used as indicators of myocardial injury were markedly increased following H/R. However, NRF2 over-expression could markedly inhibit these effects induced by H/R. Meanwhile, the elevated total cellular ROS (**Figure 1F**) and mitochondrial ROS generation (**Figure 1G**), indicated by DCFH-DA and Mito-SOX dye respectively, were significantly decreased. Concurrently, the activities of the antioxidative cytokines SOD and GSH were evaluated in all groups. Compared with the sham group, there was a significant decrease in the levels of SOD (**Figure 1H**) and GSH (**Figure 1I**) in the H/R group. As expected, compared with the H/R group, the activities of SOD and GSH were higher in the NRF2 over-expression group. These results demonstrate that NRF2 overexpression impairs the H/R induced mitochondrial ROS generation in adult cardiomyocytes.

### Keap-NRF2 signaling enhances the mitochondrial bioenergetics and NADPH oxidase activity in H/R injured adult cardiomyocyte

**Figure 2:**
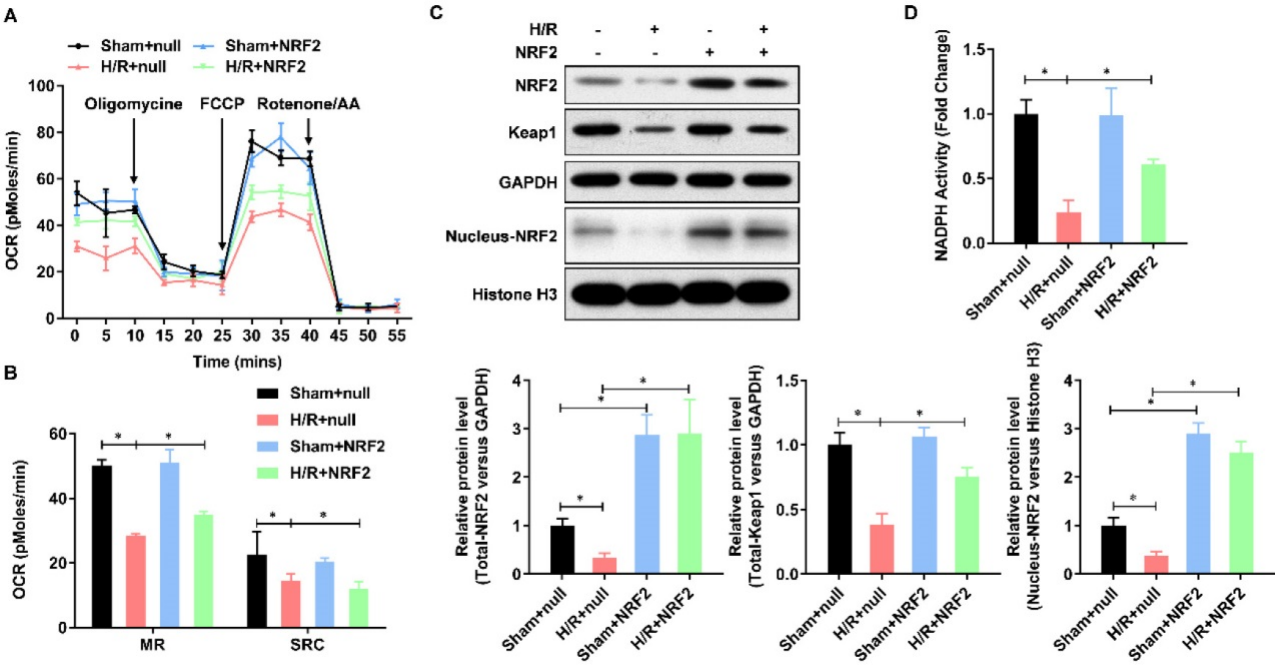
** Keap-NRF2 signaling enhances the mitochondrial bioenergetics and NADPH oxidase activity in H/R injured adult cardiomyocyte.** (A) The mitochondrial bioenergetics was measured by the Seahorse XFp Extracellular Flux Analyzer; (B) The maximal respiration (MR) and spare respiratory capacity (SPR) were analyzed; (C) The protein level of total Keap1, total NRF2 and neclues NRF2 were analyzed by Western Blot; (D) The activity of NADPH oxidase was analyzed by ELISA kit. N=3;*P<0.05, **P<0.01 verses indicated group.

Mitochondrial dysfunction plays a central role in myocardial ischemia/reperfusion (I/R) injury. Impaired electron transport chain activity and abnormal mitochondrial bioenergetics are considered to be the major contributing factors to mitochondrial dysfunction in myocardial I/R injury. To identify whether NRF2 has effect on the mitochondrial bioenergetics, we analyzed the mitochondrial respiration by detecting the rate of oxygen consumption with the Seahorse XFp Extracellular Flux Analyzer (**Figure 2A**). The oxygen consumption rate of adult cardiomyocytes, including maximal respiration (MR) and spare respiratory capacity (SPR), decreased evidently in H/R group, whereas enhanced by NRF2 (**Figure 2B**). The Keap1-NRF2 regulatory pathway plays a central role in protecting cells from oxidative damage. We then detected the protein level of Keap1, total NRF2 and nucleus NRF2 by Western Blot. As shown in **Figure 2C**, the protein level of Keap1 and total NRF2 was significantly decreased by H/R injury, whereas upregulated by NRF2 over-expression (**Figure 2C**). Moreover, the nucleus NRF2 protein level was also upregulated (**Figure 2C**), which demonstrated an activation of Keap1-NRF2 signaling in the NRF2 over-expression. Considering Keap1-Nrf2 pathway also regulates both mitochondrial and cytosolic ROS production through NADPH oxidase, we then evaluated the NADPH activity and found an obvious decrease in the H/R injured adult cardiomyocytes. However, NRF2 over-expression significantly attenuated this decreasing in the H/R injured adult cardiomyocytes (**Figure 2D**). These results collectively demonstrate that Keap-NRF2 signaling enhances the mitochondrial bioenergetics and NADPH oxidase activity in H/R injured adult cardiomyocyte.

### NRF2 is transcriptional activated by RBP-Jκ in adult cardiomyocytes

**Figure 3:**
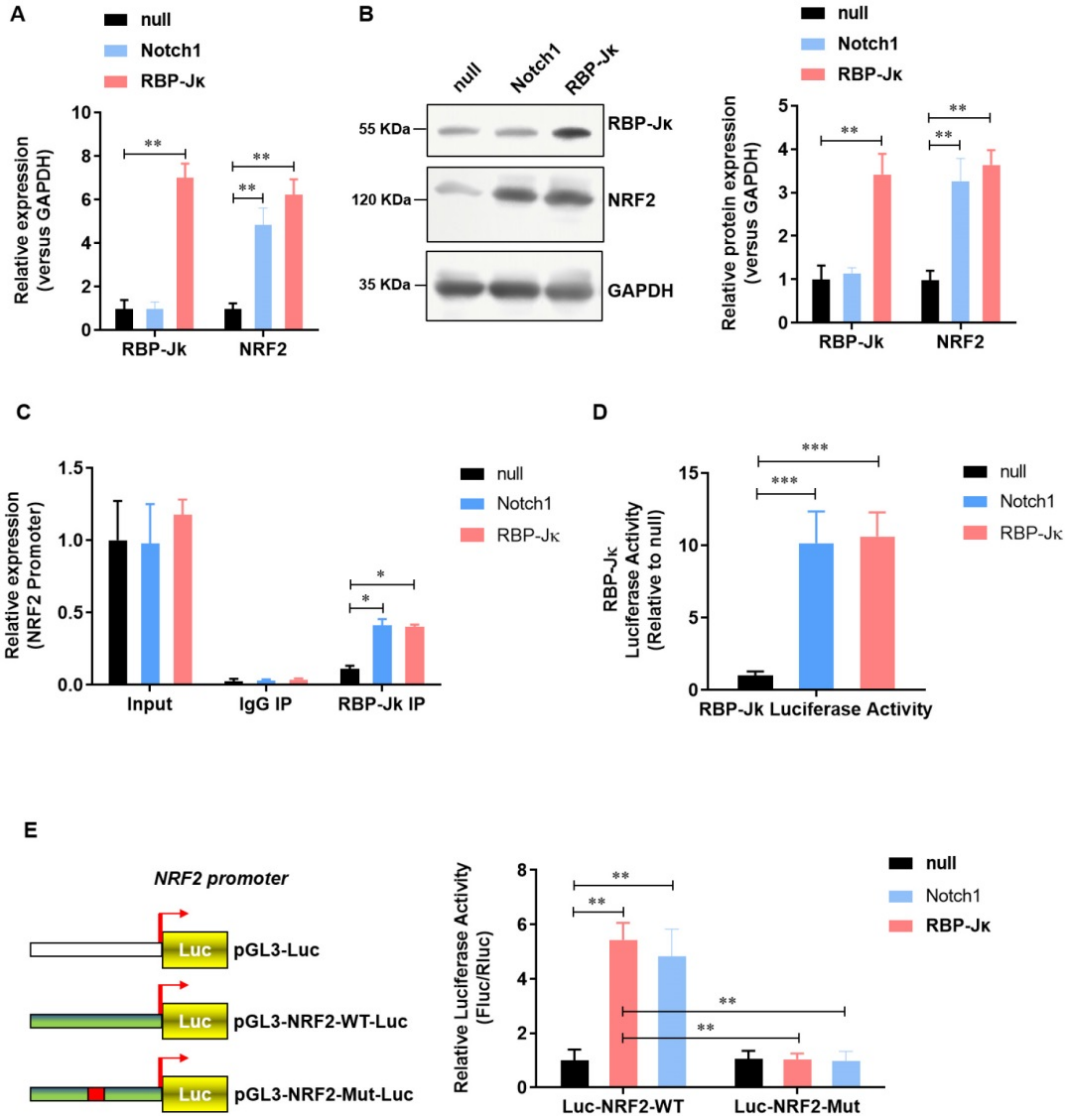
** NRF2 is transcriptional activated by Notch1/RBP-Jκ signaling in adult cardiomyocytes.** (A) The mRNA level of NRF2 was analyzed by real-time PCR; (B) The protein level of NRF2 was confirmed by Western Blot; (C) Chromatin immunoprecipitation assay was used to analysis the interaction of RBP-Jκ on the promoter region of NRF2; (D) RBP-Jκ luciferase reporter activity assay was perfomed to ananlyzed the transcriptional activity of RBP-Jκ; (E) Dual-luciferase assay was used to analysis the promoter activity of NRF2. N=3; *P<0.05, **P<0.01 verses indicated group.

We previously reported that Notch1 activates Keap1-Nrf2 signaling pathway and significantly reduce the formation of reactive oxygen species in neonate rat myocardial cells. To further illustrate the regulatory mechanism of Notch1 on Keap1-Nrf2 signaling pathway. We analyzed the effect of RBP-Jκ, a critical down-stream transcript factor of Notch1, on the expression of NRF2. Notch1 did not affect the expression level of RBP-Jκ(**Figure 3A and B**). However, both Notch1 and RBP-Jκ over-expression significantly up-regulated the expression level of NRF2 mRNA (**Figure 3A**) and protein (**Figure 3B**). Chromatin immunoprecipitation assay showed that both Notch1 and RBP-Jκ over-expression significantly improve the enrichment of RBP-Jκ in the promoter region of NRF2 (**Figure 3C**). RBP-Jκ luciferase reporter activity assay further confirmed that the transcriptional activity of RBP-Jκ was elevated by Notch1 or RBP-Jκ over-expression (**Figure 3D**). Moreover, dual-luciferase assay of the activity of NRF2 promoter suggested that RBP-Jκ significantly enhance the activity of wild-type NRF2 promoter, but not the RBP-Jκ binding site mutant NRF2 promoter (**Figure 3E**). These data suggest that NRF2 is transcriptional activated by RBP-Jκ in adult cardiomyocytes.

### Notch1 impairs the Keap1-Cullin3 interaction in adult cardiomyocytes

**Figure 4:**
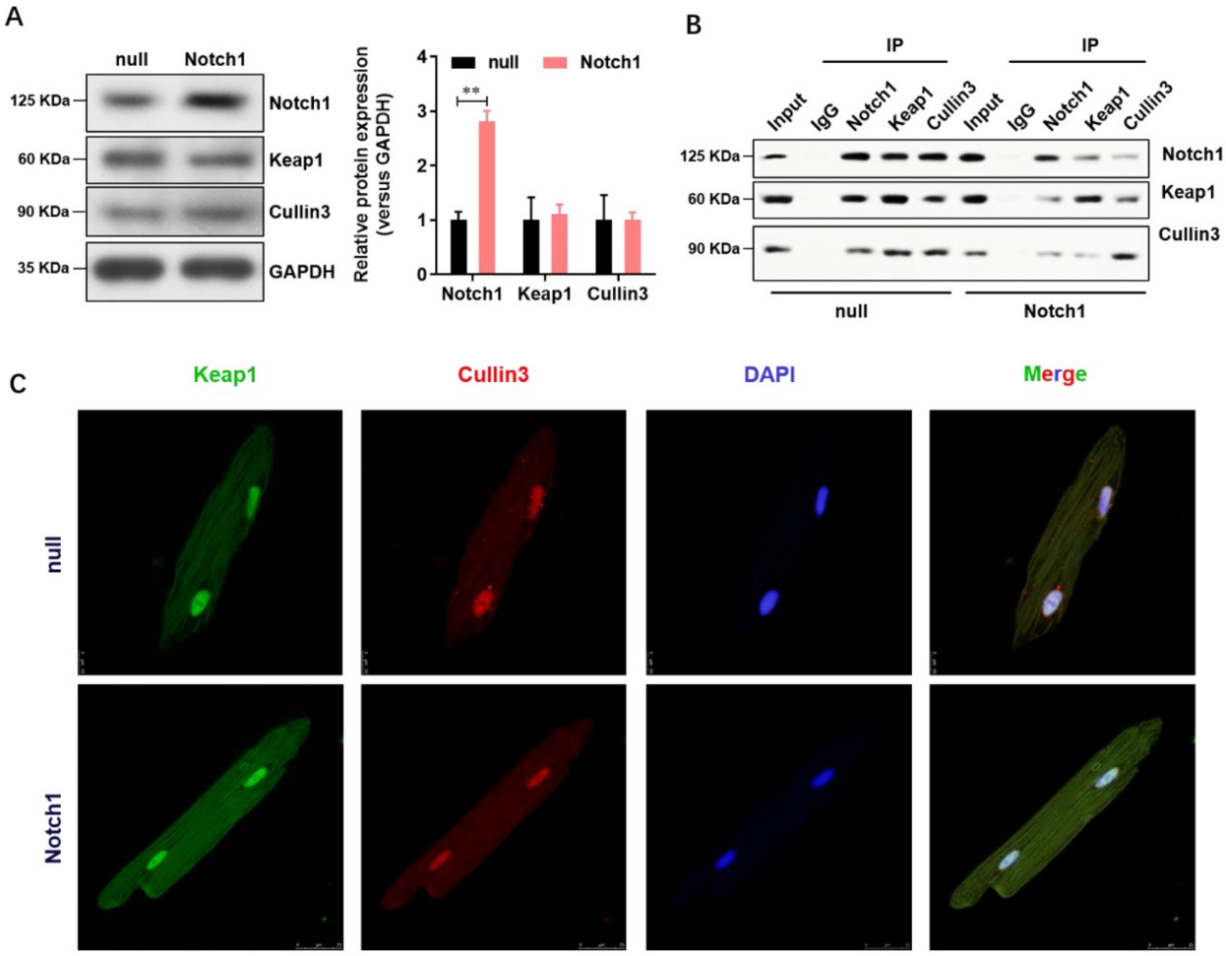
** Notch1 impairs the Keap1-Cullin3 interaction in adult cardiomyocytes.** (A) The protein level of Notch1, Keap1 and Cullin3 were confirmed by Western Blot; (B) Co-immunoprecipitation assay was used to analysis the interaction between Notch1, Keap1 and Cullin3. (C) Immunofluorescence observation of the distribution of Keap1 and Cullin3 by confocal microscopy. N=3.

Keap1 is an adaptor protein for cullin3-based ubiquitin E3 ligase and negatively regulates NRF2. To understand whether Notch1 over-expression regulate the NRF2 degradation through Keap1- Cullin3, we firstly analyzed the expression level of Keap1 and Cullin3 in normal or Notch1 over-expression adult cardiomyocytes. As shown in **Figure 4A**, Notch1 over-expression did not affect the protein level of Keap1 and Cullin3. However, Notch1 over-expression significantly impairs the protein interaction between Keap1 and Cullin3 by Co-immunoprecipitation (**Figure 4B**). This was further confirmed by immunofluorescence observation of the distribution of Keap1 and Cullin3 by confocal microscopy (**Figure 4C**). Notably, RBP-Jκ over-expression did not affect the protein interaction between Keap1 and Cullin3 (**Supplementary Figure 1**). These results showed that Notch1 impairs the Keap1-Cullin3 interaction in adult cardiomyocytes.

### NRF2 is critical to the Notch1 impaired mitochondrial ROS generation in H/R injured adult cardiomyocytes

**Figure 5:**
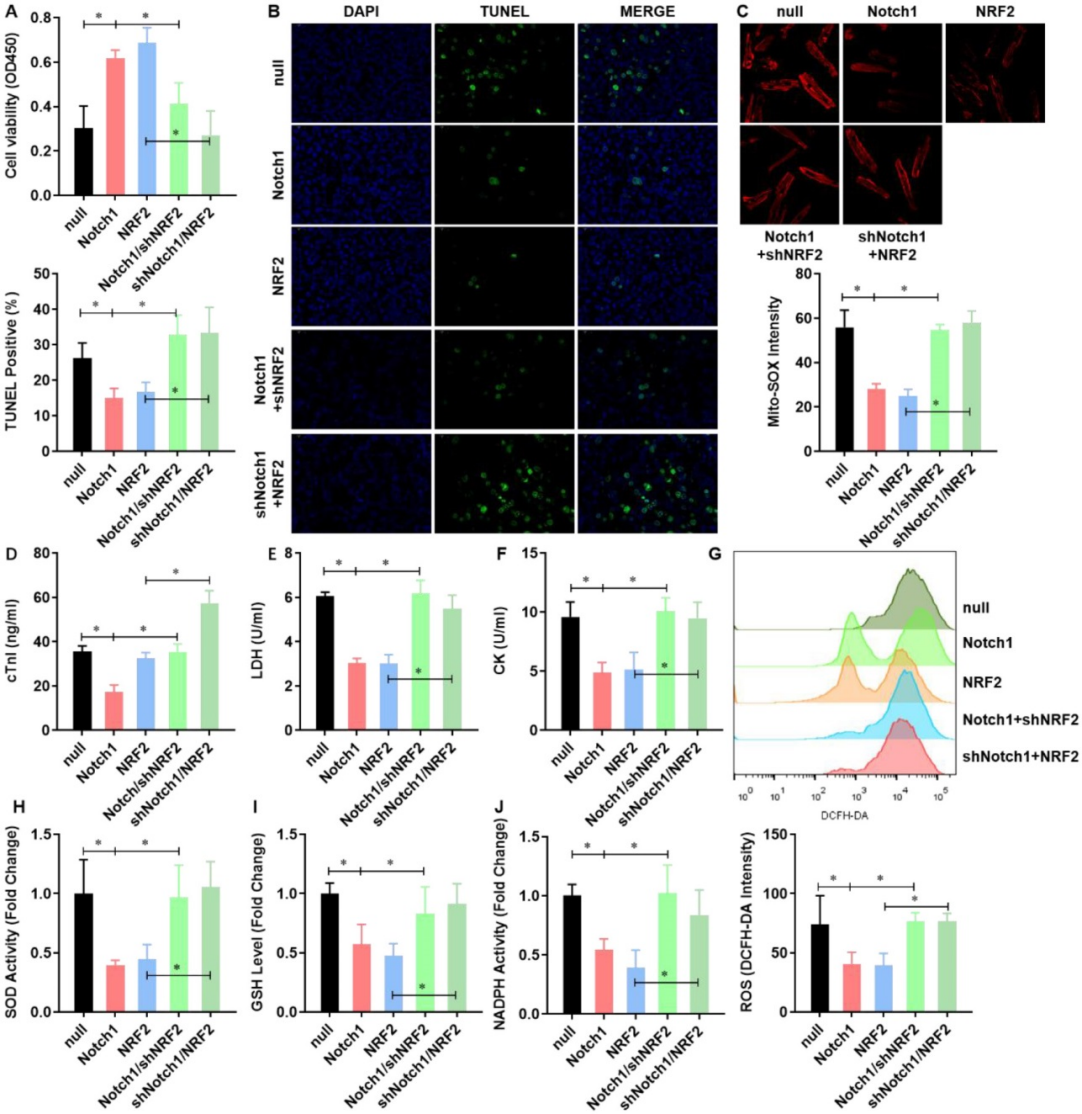
** NRF2 is critical to the Notch1 impaired mitochondrial ROS generation in H/R injured adult cardiomyocytes.** (A) The cellular viability was detected by CCK-8 assay; (B) The percentage of apoptotic cardiomyocytes was analyzed by TUNE staining; (C) The level of cTnI in the culture medium were also detected at the end of the reperfusion to assess myocardial injury; (D) The activity of LDH and (E) CK were evaluated by Electrochemiluminescence immunoassay; (F) The total cellular and (G) mitochondrial ROS were analyzed by DCFH-DA and Mito-SOX assay; (H) The levels of SOD (I) GSH and (J) the activity of NADPH oxidase were analyzed by ELISA Kit. N=3; *P<0.05, **P<0.01 verses indicated group.

To verified Notch1 attenuated the mitochondrial ROS generation in H/R injured adult cardiomyocytes via regulating NRF2, we performed a rescue assay in adult cardiomyocytes with Notch1 or NRF2 over-expression/knock-down. Both Notch1 and NRF2 improved the cell viability of H/R injured adult cardiomyocytes (**Figure 5A**). However, NRF2 knock-down obviously decreased the cell viability in Notch1 over-expressed H/R adult cardiomyocytes (**Figure 5A**). Interestingly, Notch1 knock-down also decreased the cell viability in NRF2 over-expressed H/R adult cardiomyocytes (**Figure 5A**). Similar phenotype was observed in the percentage of apoptotic H/R adult cardiomyocytes (**Figure 5B**) and serious myocardial damage markers, including cTnI (**Figure 5C**), LDH (**Figure 5D**) and CK (**Figure 5E**). Moreover, the Notch1/NRF2 over-expression attenuated total cellular ROS (**Figure 5F**) and mitochondrial ROS generation (**Figure 5G**) were significantly elevated by NRF2 or Notch1 knock-down respectively. The activities of the antioxidative cytokines SOD (**Figure 5H**), GSH (**Figure 5I**) and the activity of NADPH oxidase (**Figure 5J**) were also inhibited by NRF2 or Notch1 knock-down in Notch1/NRF2 over-expressed H/R adult cardiomyocytes respectively.

### Keap-NRF2 signaling contributes to the Notch1 protected mitochondrial bioenergetics and NADPH oxidase activity in H/R injured adult cardiomyocyte

**Figure 6:**
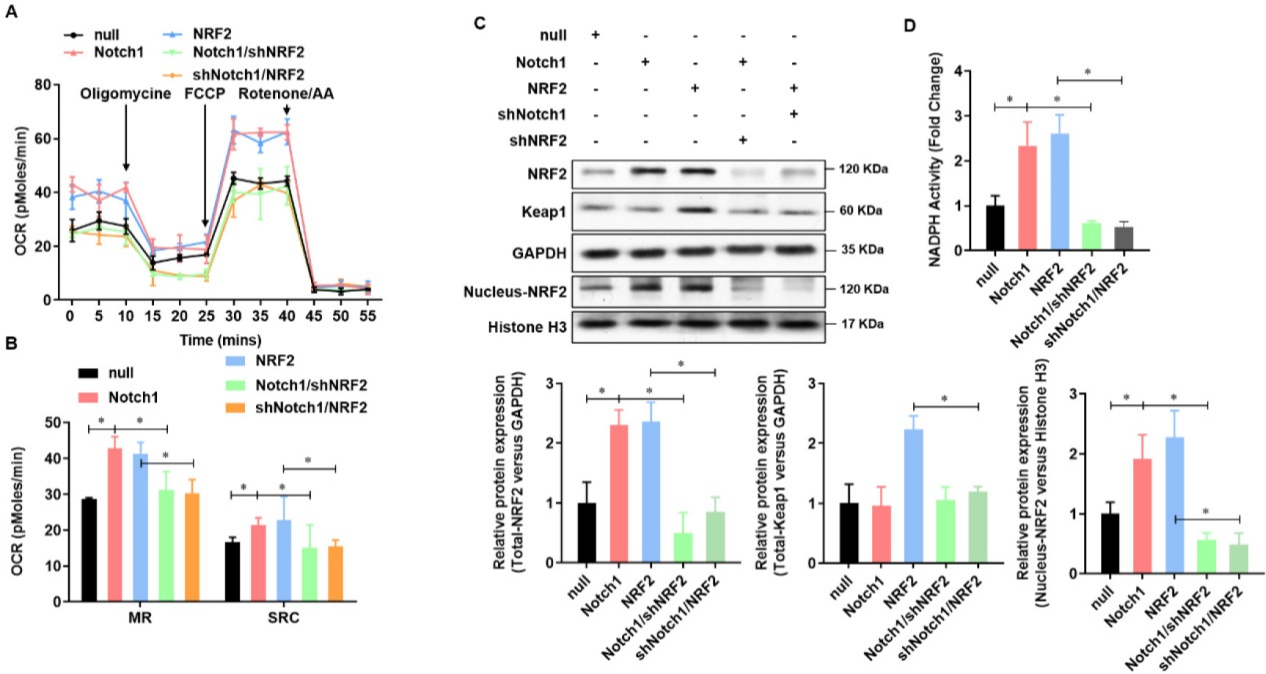
** Keap-NRF2 signaling contributes to the Notch1 protected mitochondrial bioenergetics and NADPH oxidase activity in H/R injured adult cardiomyocyte.** (A) The mitochondrial bioenergetics was measured by the Seahorse XFp Extracellular Flux Analyzer; (B) The maximal respiration (MR) and spare respiratory capacity (SPR) were analyzed; (C) The protein level of total Keap1, total NRF2 and neclues NRF2 were analyzed by Western Blot; (D) The activity of NADPH oxidase was analyzed by ELISA kit; *P<0.05, **P<0.01 verses indicated group. N=3.

To further verified Notch1 protected mitochondrial bioenergetics and NADPH oxidase in H/R injured adult cardiomyocytes via regulating NRF2, we then analyzed the mitochondrial respiration in adult cardiomyocytes with Notch1 or NRF2 over-expression/knock-down (**Figure 6A**). The oxygen consumption rate of adult cardiomyocytes, including maximal respiration (MR) and spare respiratory capacity (SPR), increased by Notch1 or NRF2 over-expression, whereas decreased by NRF2 or Notch1 knock-down (**Figure 6B**). On the other hand, the protein level of Keap1 was elevated by NRF2 over-expression, but this increasing was abolished by Notch1 knock-down. However, the protein level of Keap1 was not affect by Notch1 over-expression (**Figure 6C**). Meanwhile, the protein level of NRF2 was increased by either Notch1 or NRF2 over-expression, whereas decreased to normal level by NRF2 or Notch1 knock-down (**Figure 6C**). Furthermore, Notch1 or NRF2 over-expression significantly attenuated the NADPH activity decreasing in the H/R injured adult cardiomyocytes, which was abrogated by NRF2 or Notch1 knock-down (**Figure 6D**). These results suggested that Keap-NRF2 signaling contributes to the Notch1 protected mitochondrial bioenergetics and NADPH oxidase activity in H/R injured adult cardiomyocyte.

### Keap1-NRF2 signaling is critical to the Notch1 protected myocardial systolic function and impaired tissue injury

**Figure 7:**
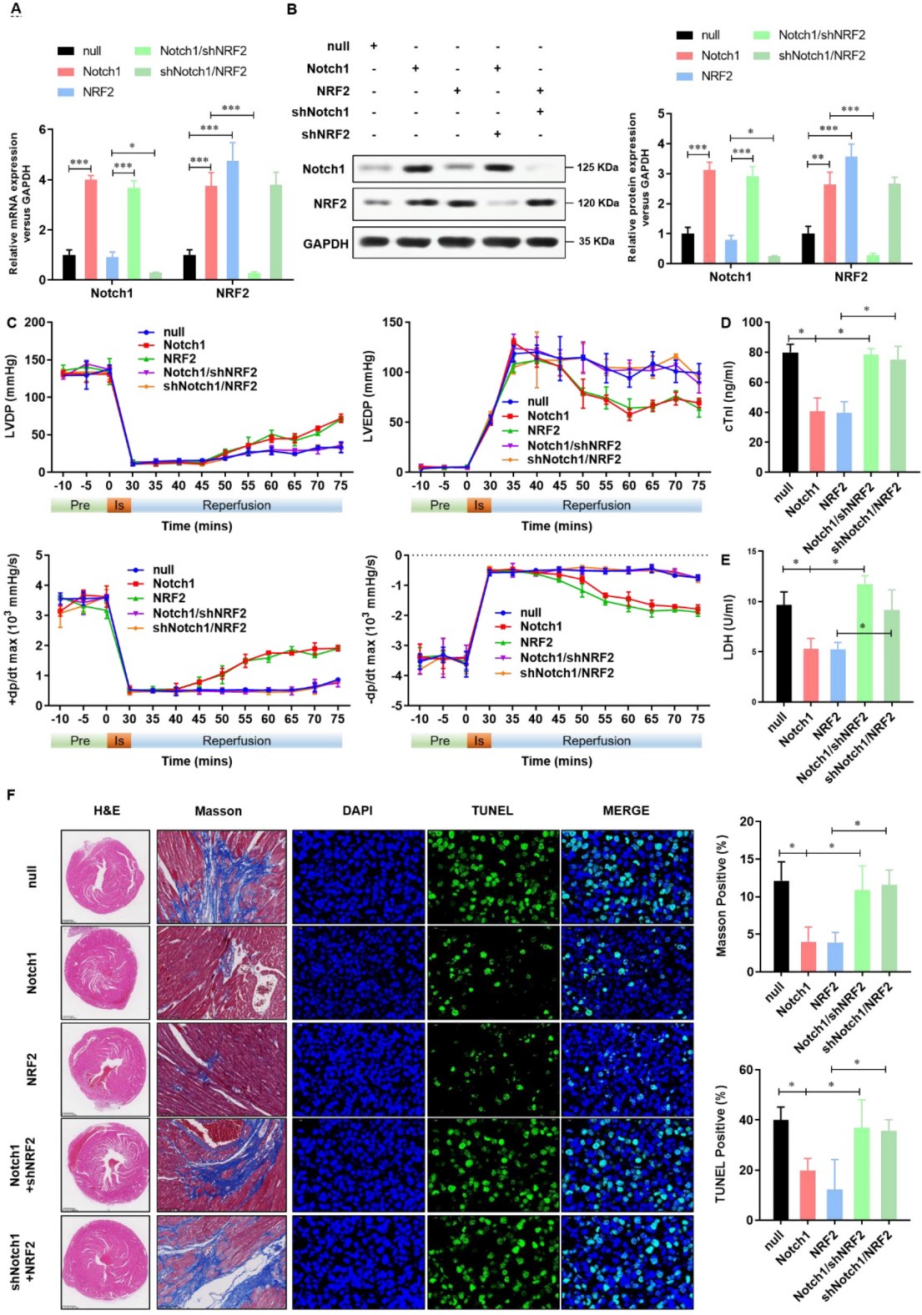
** Keap1-NRF2 signaling is critical to the Notch1 protected myocardial systolic function and impaired tissue injury.** (A) The mRNA expression of Notch1 and NRF2 were analyzed by real-time PCR; (B)The protein expression of Notch1 and NRF2 were analyzed by Western Blot; (C), Representative traces and summarized data of LV pressure (LVP) during ischemic-reperfusion (I/R) in isolated rat hearts from indicated groups; (D) The level of cTnI was detected at the end of the reperfusion to assess myocardial injury; (E) The activity of LDH was evaluated by Electrochemiluminescence immunoassay; (F) Representative pictures of H&E-stained, Masson trichrome stained and TUNEL stained cardiac sections were shown. N=5; *P<0.05, **P<0.01 verses indicated group.

To confirm the cardioprotective effect of Notch1-Keap1-NRF2 signaling in vivo, as shown in **Figure 7A and 7B**, we over-expressed Notch1/NRF2 with or without NRF2/ Notch1 knock-down and analyzed the postischemic contractile function in Langendorff-perfused rat hearts. The preischemic contractile parameters were similar between the five groups, while the I/R (30 min/45 min)-suppressed LV contractile function, characterized by LV develpment pressure (LVDP), LV end-diastolic pressure (LVEDP), and maximal speed of LV pressure development and decline (±dp/dt), was markedly alleviated by Notch1/NRF2 over-expression (**Figure 7C**). However, with the knock-down of NRF2 or Notch1, the cardioprotective effect of Notch1 or NRF2 over-expression on the contractile function were totally abrogated (**Figure 7C**). Consistently, I/R-induced cTnI level (**Figure 7D**) and LDH activity (**Figure 7E**) in coronary perfusate were significantly inhibited by Notch1 or NRF2 over-expression, whereas dramatically impaired by the knock-down of NRF2 or Notch1 respectively. Moreover, the degree of damaged myocardium and fibrotic scar tissue was determined. As shown in **Figure 7F**, HE staining indicated that the structure of myocardial tissue in the I/R group, the cardiomyocytes arrangement was messy, and the cardiomyocytes were enlarged. Compared with the I/R group, Notch1 and NRF2 over-expression had a clear myocardial tissue structure and lighter myocardial damage. In the Notch1/shNRF2 and shNotch1/NRF2 groups, the myocardial tissue was aggravated compared to the Notch1 or NRF2 over-expression groups but reduced compared to the I/R group. Mason staining was further used to detect myocardial collagen deposition in all groups. Collagen deposition in I/R group was obvious presented (**Figure 7F**). Compared with I/R group, the collagen fibers accumulation between cardiomyocytes was significantly reduced in the Notch1 or NRF2 over-expression group (**Figure 7F**). In comparison, the positive staining of myocardial collagen in the Notch1/shNRF2 and shNotch1/NRF2 groups was significantly higher than that in the Notch1 or NRF2 over-expression group (**Figure 7F**). Furthermore, Similar phenotype was observed in the percentage of apoptotic I/R LV tissues (**Figure 7F**). Collectively, these results demonstrates that Keap1-NRF2 signaling is critical to the Notch1 protected myocardial systolic function and impaired tissue injury.

## Discussion

Coronary heart disease is a major cause of death worldwide, and its pathogenesis has been studied for decades^14^-^20^. A series of studies have shown that Notch1 protects the heart from I/R damage, but the underlying mechanism is not fully understood. We previously reported that Notch1 activates RISK/SAFE/HIF-1 alpha signal, reduces ROS in cardiomyocytes, enhances cardiomyocyte viability, improves mitochondrial fusion, and significantly reduces myocardial I/R injury^10^-^13^. Herein, we demonstrate that Notch1 impaired mitochondrial ROS generation and improves the mitochondrial bioenergetics via Keap1-NRF2 signaling pathway in I/R injured cardiomyocytes and heart.

The evolutionally conserved Notch signaling pathway controls tissue formation and homeostasis in embryos and adults through local cell-cell interactions^21^. Notch receptor (Notch1-4) is cleaved by TNF-α-transferase (TACE) and γ-secretase complexes after recognition by delta-like1,3,4 and Jagged1, 2^22^. The released Notch intracellular domain (NICD) translocate to the nucleus and binds to the transcription factor CSL (c-promoter binding factor-1/Suppressor of hairless/ LAG1) and regulates the transcription of the target genes Hes and Hey^23^,^24^. In the heart, Notch is expressed in a variety of cell types, including cardiomyocytes, smooth muscle cells, and endothelial cells^25^. Notch1 is downregulated during postnatal development, but may be activated in response to myocardial injury, suggesting that Notch signaling may have a protective role^26^. The mechanism of Notch1-mediated cardiac protection is complex and not fully understood. We have previously found that Notch1 signal is activated in the process of myocardial IPC and IPost, which can improve cell viability and inhibit apoptosis^10^. In addition, the activated Notch1 signal stabilized the mitochondrial membrane potential and reduced IRI induced reactive oxygen species. In addition, in the Langendorff cardiac perfusion model, activated Notch1 signal restores cardiac function, reduces lactate dehydrogenase release, and limits infarct size after myocardial ischemia^10^. In this study, we further found that NRF2 is a functional down-stream target of Notch1 signaling during the protection of heart.

NRF2 is one of the CNC family of transcription factors including NRF1, 2, 3, BACH1, 2, and NF-E2P45^27^. Expression of NRF2 target genes is highly and rapidly induced after cell exposure to endogenous and exogenous oxidants and electrophilic stress^28^. It is reported that Keap1-NRF2 signaling and Notch1 signaling can be regulated by reciprocal transcriptional machinery, NRF2-Notch1 crosstalk influences the expression of defense systems against endogenous and exogeneous stressors leading to cellular protection and enhances maintenance of cellular homeostasis^29^-^32^. In this study, we showed that Notch1 activated the expression of NRF2 in an RBP-Jκ dependent manner. In contrast, Notch1 inhibited NRF2 degradation via disturbing the protein interaction between Keap1 and Cullin3. However, RBP-Jκ did not affect the protein interaction between Keap1 and Cullin3. On the cardio protection effect of NRF2, we can also see that NRF2 has a similar cardio protection effect with Notch1 and NRF2 knock-down could impair the Notch1 induced effect, which suggest that NRF2 would be a critical signaling for the Notch1 acting cardio protection role. In contrast, Notch1 knock-down also impairs the NRF2 induced cardio protection effect, which suggests that Notch1 and NRF2 is not a straight signaling pathway. Notch1 is also critical to NRF2 in the cardio protection progress.

In conclusion, this study presented a detailed mechanism by which Notch1-NRF2 signaling impairs mitochondrial ROS generation and improves mitochondrial bioenergetics during myocardial protection. Further studies are needed to determine the clinical significance of these effects and to develop new therapies for patients with I/R, as treating this disease remains clinically challenging.

## Supplementary Material

Supplementary figure.
