# Supplementary material for: Keap-NRF2 signaling contributes to the Notch1 protected heart against ischemic reperfusion injury via regulating mitochondrial ROS generation and bioenergetics
Source: Int J Biol Sci. 2022 Feb 7;18(4):1651–62. doi: 10.7150/ijbs.63297 (PMC8898363; doi:10.7150/ijbs.63297)
Supplement: Supplementary file 1 — Supplementary figure. [file ijbsv18p1651s1.pdf]

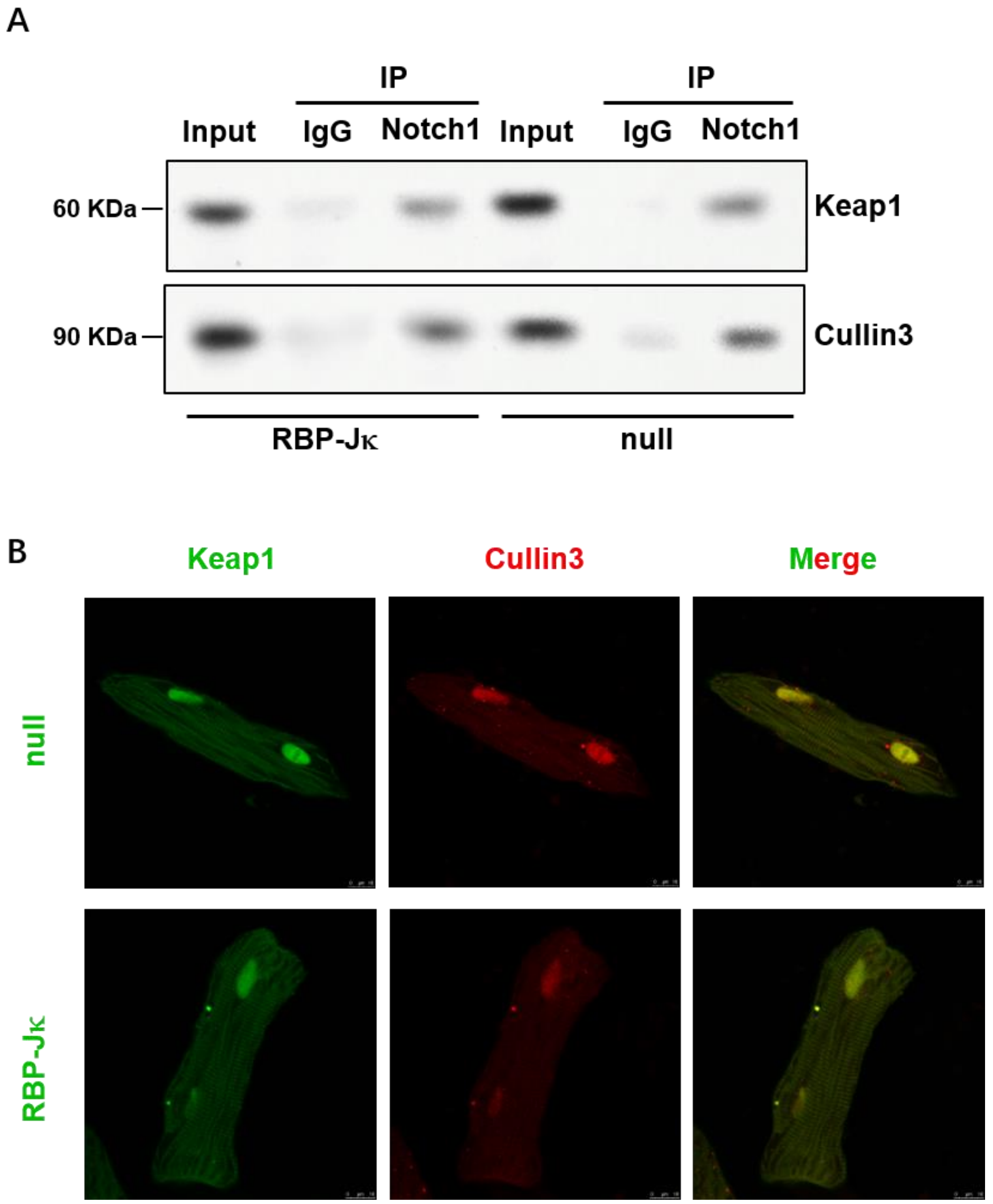

Supplementary Figure 1. RBP-Jκ do not affect the Keap1-Cullin3 interaction in adult cardiomyocytes. (A) Co-immunoprecipitation assay was used to analysis the interaction between Notch1, Keap1 and Cullin3. (B) Immunofluorescence observation of the distribution of Keap1 and Cullin3 by confocal microscopy. N=3.
